# Genetic Variability and Population Structure of *Salvia lachnostachys*: Implications for Breeding and Conservation Programs

**DOI:** 10.3390/ijms16047839

**Published:** 2015-04-08

**Authors:** Marianna Erbano, Guilherme Schnell e Schühli, Élide Pereira dos Santos

**Affiliations:** 1 Programa de Pós-Graduação em Ciências Farmacêuticas, Universidade Federal do Paraná, 80210-170 Curitiba, Paraná, Brazil; E-Mail: mariannaer@yahoo.com; 2 Embrapa Florestas, 83411-000 Colombo, Paraná, Brazil; E-Mail: schuhli@gmail.com; 3 Departamento de Botânica, Setor de Ciências Biológicas, Universidade Federal do Paraná, 81530-900 Curitiba, Paraná, Brazil

**Keywords:** intraspecific diversity, ISSR, Labiatae, Southern Brazil

## Abstract

The genetic diversity and population structure of *Salvia lachnostachys* Benth were assessed. Inter Simple Sequence Repeat (ISSR) molecular markers were used to investigate the restricted distribution of *S. lachnostachys* in Parana State, Brazil. Leaves of 73 individuals representing three populations were collected. DNA was extracted and submitted to PCR-ISSR amplification with nine tested primers. Genetic diversity parameters were evaluated. Our analysis indicated 95.6% polymorphic loci (stress value 0.02) with a 0.79 average Simpson’s index. The Nei-Li distance dendrogram and principal component analysis largely recovered the geographical origin of each sample. Four major clusters were recognized representing each collected population. Nei’s gene diversity and Shannon’s information index were 0.25 and 0.40 respectively. As is typical for outcrossing herbs, the majority of genetic variation occurred at the population level (81.76%). A high gene flow (*Nm* = 2.48) was observed with a correspondingly low fixation index. These values were generally similar to previous studies on congeneric species. The results of principal coordinate analysis (PCA) and of arithmetic average (UPGMA) were consistent and all three populations appear distinct as in STRUCTURE analysis. In addition, this analysis indicated a majority intrapopulation genetic variation. Despite the human pressure on natural populations our study found high levels of genetic diversity for *S. lachnostachys*. This was the first molecular assessment for this endemic species with medicinal proprieties and the results can guide for subsequent bioprospection, breeding programs or conservation actions.

## 1. Introduction

The main centers of diversity for the genus *Salvia* L. (Lamiaceae) are in Southwest Asia and the Americas, Central and South America [1]. In Brazil, *Salvia* is mostly found in the Midwest, Southeast and South [2]. Representatives of this genus are used in folk medicine, such as *Salvia miltiorrhiza* Bunge which is used for the treatment of cardiovascular diseases [3]. Indeed, some species of *Salvia* have clear pharmacological properties, including, anti-inflammatory [4], gastroprotective [5], antiplatelet and antithrombotic effects [6]. Essential oils and terpenoids are abundant, and represent the principal chemical components in *Salvia* species [7].

One of the most geographically restricted species is *Salvia lachnostachys* Benth., a perennial native herb found mainly in Paraná State, southern Brazil [8,9]. Studies with the essential oil from the flowers and leaves of this species indicate the presence of saturated aliphatic compounds and a sesquiterpene fraction [10]. In the hexane fraction of dried, minced leaves, two hydrated triterpene acids (ursolic acid and oleanolic acid) and a norditerpene called fruticulin A were found [9]. The ethanolic extract and fruticulin A from the leaves of *Salvia lachnostachys* showed have anti-inflammatory and the analgesic effects in oedema, pleurisy, and hyperalgesia induced by carrageenan models in mice [11].

One of the most common methods to study genetic structure and variance in geographically restricted species such as *S. lachnostachys* is through molecular markers. A commonly used technique is inter-simple sequence repeats (ISSRs), which employs the polymerase chain reaction (PCR) and microsatellite sequences to generate multilocus markers with a high degree of reproducibility [12]. Some of the advantages related to this technique include low costs and the possibility of implementation without prior knowledge of the plant genome [13,14]. Moreover, ISSR is accessible to most molecular biology labs and, therefore, has been widely employed in the analysis of genetic variability. Several studies using this technique have been published generally aimed at informing conservation actions [15,16], or for pharmacognostics and authentication [17,18].

Given the dramatic and ongoing threats to Brazil’s wildlife and natural landscapes [19], there is an urgent need to assess the diversity and viability of natural populations, especially those that have economic potential. In this context, *S. lachnostachys* is a prime candidate for analysis of population level genetic variability, being a native species with great medicinal potential and a highly restricted geographic distribution.

## 2. Results

**Figure 1:**
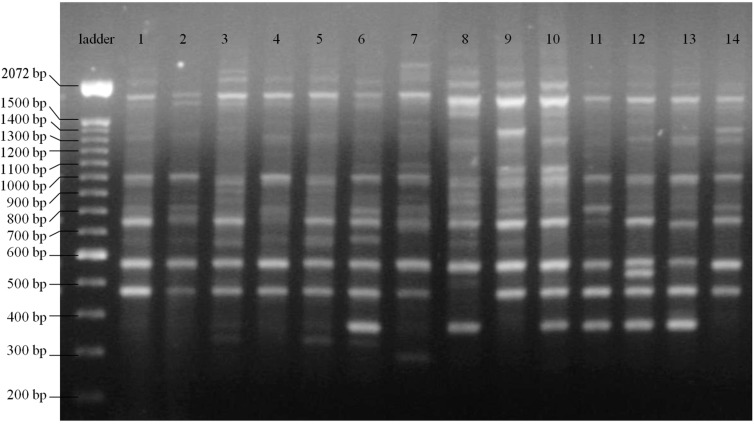
Inter Simple Sequence Repeat (ISSR) profiles of *Salvia lachnostachys* Benth. collected in Paraná State, Brazil, obtained with primer (CTC)_4_RC, first lane: marker of 100 bp DNA ladder (Invitrogen), *lanes 1–8* from Curitiba; *9–14* from São Luiz do Purunã.

The ISSR analysis produced 10 to 22 amplified bands per primer with an average of 17.6 polymorphic fragments (Figure 1, Table 1). Out of the 159 amplified bands, 152 were polymorphic. The scored fragment sizes ranged from 250 to 2200 bp. Estimates of precision based on 159 loci revealed high correlation values reaching 0.39 with 10 loci, 0.87 with 80 and 0.99 with 150. The stress value with 150 loci was 0.02 (values under 0.05 indicates good sample sufficiency based on Kruskal’s goodness-of-fit index). The percentage of polymorphic bands ranged from 86% to 100%, (95.6% average). Simpson’s index of each primer ranged from 0.66 to 0.86 (0.79 average). In general, dinucleotide repeats (AG)_8_YC and (AG)_8_A, showed the highest polymorphism.

**Table 1:** ISSR primers used in the analysis of *Salvia lachnostachys* Benth. (Lamiaceae).

Sequence (5'→3')	AT (°C)	NB	NPB	PPB (%)	Simpson’s Index	Reference
(AC)_8_T	51.4	14	13	93	0.79	[20]
(AG)_8_A	46.7	12	11	92	0.84	[20,21]
(AG)_8_C	48.8	20	20	100	0.83	[22]
(AG)_8_YC	50.2	22	22	100	0.86	[20]
(AG)_8_YT	49.2	19	19	100	0.77	[21,22]
(CA)_8_G	51.0	19	19	100	0.78	[20,21]
(CT)_8_A	44.7	10	10	100	0.66	[21]
(CTC)_4_RC	51.7	22	19	86	0.77	[20,21]
(GA)_8_T	45.4	21	19	90	0.80	[20,21,22]
Total	-	159	152	96	0.79	-
Average	-	17.6	-	-	-	-

AT = annealing temperature; NB = numbers of bands; NPB = numbers of polymorphic bands; PPB = percentage of polymorphic bands; Y = C or T, R = A or G.

**Figure 2:**
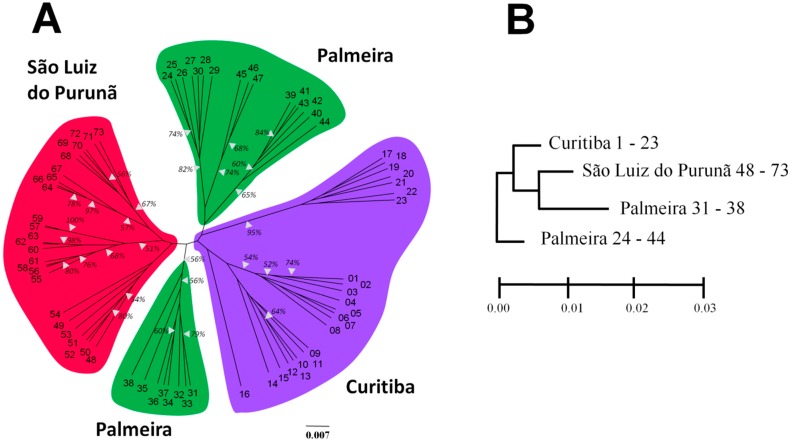
Genetic relationships of three populations of *Salvia lachnostachys* Benth. based on ISSR markers. (**A**) Unweighted Pair-group Method using Arithmetic average (UPGMA) unrooted tree illustrating genetic relationships among 73 individuals of *S. lachnostachys* analyzed with 159 loci obtained with PCR/ISSR. The geographical origin is indicated by different colors. Bootstrap support percentages of higher than 50% are given (10,000 replicates); (**B**) Collapsed UPGMA tree depicting relationships among populations. Samples included are indicated after population name.

**Figure 3:**
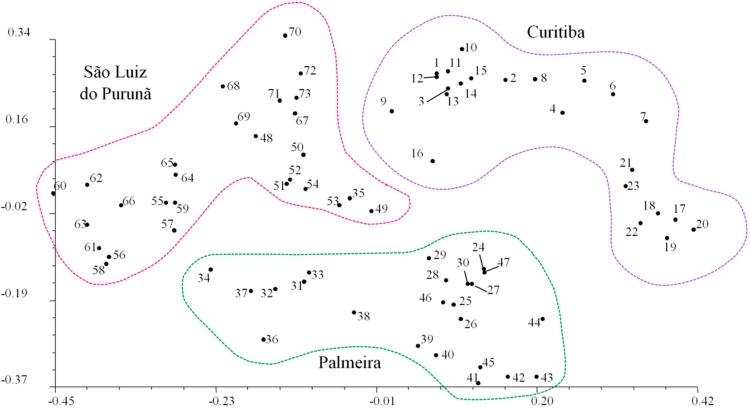
Principal components analysis from 73 individuals of *S. lachnostachys* collected on three locations in the Parana State, Brazil.

Cluster analysis generated a tree (Figure 2A) and a dendrogram (Figure 2B) in which the geographical precedence of each sample is falls into one of four major clusters. The topology suggests a group with individuals from Curitiba (individuals 1–23); Palmeira (31–38 and 24–44); and São Luiz do Purunã (48–73). The genetic relationship of individual accessions was analyzed using principal component analysis (PCA). PCA analysis (Figure 3) allowed the identification of the geographical origin of the respective populations.

A summary of the ISSR data from each population of *S. lachnostachys* are given in Table 2. The percentage of polymorphic bands ranged from 69.81% to 81.76%, (97.48% average). The *observed number* *of alleles per locus* (*Ao*) and *expected number of alleles* (*Ae*) were 1.70 to 1.82 and from 1.33 to 1.39, respectively. *Nei’s gene diverstity* (*H*) and *Shannon’s information index* (*I*) of different geographic groups ranged from 0.20 to 0.24 and from 0.31 to 0.37, respectively. The group of samples from Curitiba showed the highest genetic variability while the group from São Luiz do Purunã showed the lowest.

The analysis of molecular variance (AMOVA) indicated that the majority of genetic variation occurred at the population level. The genetic differentiation among populations was 16.8% (*G_st_* = 0.1678), and within populations was 81.76%. Gene flow (*Nm* = 2.48) indicated low genetic differentiation among populations, and therefore, corroborates a low value for *F_st_* = 0.18.

**Figure 4:**
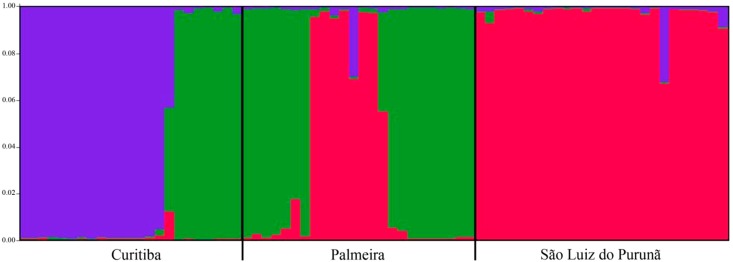
Estimated geographic group structure for three populations of *Salvia lachnostachys* Benth. with ISSR markers. Different greyscale colors indicate different geographic groups (Palmeira, São Luiz do Purunã, Curitiba). *Long black lines* indicate the separation among a priori assigned groups.

*S. lachnostachys* in the study populations has a heterogeneous genetic structure (Figure 4). The Palmeira population is the most variable among the three populations, while the Curitiba and São Luiz do Purunã populations are the most conservative.

**Table 2:** Genetic diversity of three populations of *Salvia lachnostachys* Benth. (Lamiaceae).

Populations	N	M	NPB	PPB	Ao	Ae	H	I
Curitiba	23	23	130	81.76	1.82	1.39	0.24	0.37
Palmeira	24	23	121	76.10	1.76	1.33	0.21	0.33
São Luiz Purunã	26	25	111	69.81	1.70	1.33	0.20	0.31
Total	73	70	155	97.48	1.97	1.39	0.25	0.40

N = numbers of individuals; M = numbers of polymorphic individuals; NPB = numbers of polymorphic bands; PBP = % polymorphic bands; Ao = observed number of alleles; Ae = expected number of alleles; H = Nei’s gene diversity; I = Shannon’s information index.

## 3. Discussion

The chemical composition of *Salvia* strongly indicates that the herb has potential to become an important raw material for anti-inflammatory compounds and knowledge of the diversity of wild populations will therefore be important to inform the use and conservation of this genus. Genetic surveys, in particular, are key measures to efficiently access the genetic resources of species of pharmacological interest.

Several markers have been previously applied to survey genetic variability within the genus *Salvia* [23,24,25]. Specifically, there are some important publications addressing *S. miltiorrhiza*, most of them utilizing dominant markers [23,24,25,26,27].

Despite the increasing human pressure on natural areas in Brazil, the *Salvia* species (*S. lachnostachys*) used in our study had high levels of genetic variability (PPB = 97.48%, I = 0.3982). This result suggests that the species is relatively resilient, and provides a good opportunity to study, collect and conserve *S. lachnostachys* diversity, including the use of germplasm banks. Such actions would reduce the diversity loss and genetic erosion found in cultivated species, which typically have lower diversity associated with human assisted propagation and germplasm exchange [23,28].

When considered alongside the Simpson index, the sampling variance values suggest an acceptable level of precision using the selected primers (Table 2). Based on the level of polymorphism detected, these primers may therefore represent a useful starting point for further population studies in *Salvia*. Nevertheless, the inclusion of a greater number of markers would presumably further increase the value of the Simpson index.

Wild populations of *S. miltiorrhiza* were previously evaluated using AFLPs [29]. The average Nei’s gene diversity index found (H = 0.2612) was very consistent with that observed in the present evaluation of wild *S. lachnostachys* (H = 0.2509). Comparisons with a cultivated population of *S. miltiorrhiza* reveal a lower average value of Nei’s gene diversity index (H = 0.1951) [23]. However, it is important to remember for these comparisons that Nei’s gene diversity index is an estimate of the expected heterozygosity. In the current study, considering a dominant marker, the gene diversity is derived from the calculated expected allele frequencies. Thus, the index is somewhat limited and its precision relies on the validity of the Hardy-Weinberg assumption. For this reason, comparisons of Nei’s gene diversity values from studies using different marker classes should not be made directly. For both previously cited studies in *Salvia* [23,29], dominant markers were used (RAPD and ISSR respectively). Another issue related to comparisons of Nei’s gene diversity is that different markers have different abilities to recover polymorphisms. For example, many authors have noted that ISSR markers detect higher number of polymorphic loci than isozymes and RAPD methods [28,30,31]. Therefore, the index should be considered an indicative, and comparisons should always consider these differences. The Shannon diversity index values are consistent with the population and species level genetic variability.

The unrooted dendrogram obtained in the cluster analysis effectively discriminated the sampled localities in distinct clusters based on Nei and Li distances. The dendrogram suggests a consistent differentiation among the sampled populations. The high genetic variability maintained within populations is encouraging. However, the division of samples from Palmeira in two distinct clusters was not expected; this may be related to many causes, such as founder effects or restricted gene flow. The value *G_st_* in *S. lachnostachys* was 0.1678 indicating that greater variation exists within populations than among populations. This result is consistent with that found for *S. miltiorrhiza* (*G_st_* = 0.1336). Many factors contribute to genetic structure in plant populations, especially reproductive biology, gene flow, seed dispersal and nature selection. In particular, reproductive biology plays a central role in the genetic structure [32]. It is conceivable that the outcrossing strategy previously recorded for *Salvia* [33] is responsible for the higher observed diversity within populations. This is particularly probable with high population densities where outcrossing rates are higher.

The high adaptability and wide distribution of *S. lachnostachys* in the study area may also contribute to the low levels of differentiation between populations. Specifically, a high number of migrants (*Nm*) may facilitate gene exchange between populations. Vegetative reproduction (sprouting) may also reintroduce genes after an extinction event (e.g., fire) in a single sub-population. However, the most important mechanism promoting gene flow in natural populations of plants is through the spread of seeds and pollen [34] and it is possible that prevailing winds connect the populations in the study areas. Moreover, there are several main roads crossing these areas that may act as corridors for insects, the main pollinators of *Salvia* species. The exchange of genetic material has been observed in other *Salvia* species; the *Nm* in *S. miltiorrhiza* was 3.2362 [23]. This value is similar to that observed in the present study (2.4806). This level of migration may be sufficient to partially counteract the genetic differentiation caused by genetic drift within single populations explaining the low differentiation among them [35]. Nevertheless, the results generated from Structure for all the populations indicated a value of K = 3, indicating that it is possible to distinguish three genetically different groups with genotypes attributed according to geographical origin. Structure assignment helped to clarify the topology found in UPGMA unrooted dendrogram. Samples 17 to 23 (collected in Curitiba) were indicated by the Bayesian admixture analysis as similar to genotypes collected from Palmeira and samples 31–38 (collected in Palmeira) were indicated as similar to genotypes from São Luiz do Purunã supporting the perceived pattern of high gene exchange and low genetic differentiation among populations.

## 4. Experimental Section

### 4.1. Sampling and DNA Extraction

**Figure 5:**
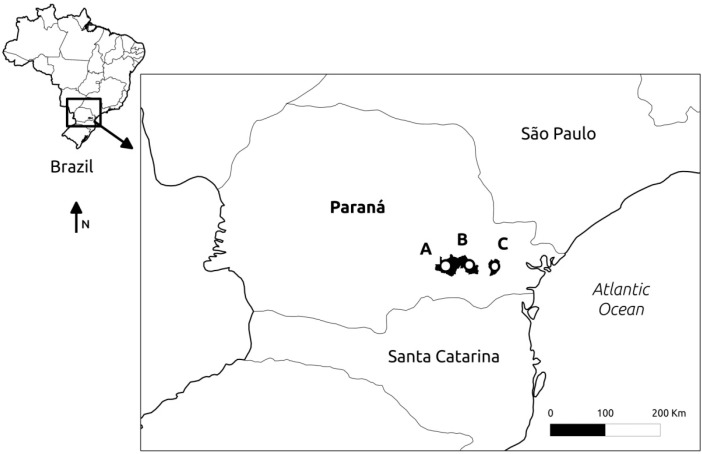
Locations of the different populations of *Salvia lachnostachys* Benth. in Paraná State, Brazil; (A) Palmeira; (B) São Luiz do Purunã; (C) Curitiba.

Field sampling covered the restricted distribution of *S. lachnostachys* in Parana State, southern Brazil. The geomorphological profile for this State consists of an Atlantic coastal plain at its eastern limits followed by a mountain ridge and three consecutive plateaus corresponding, respectively, to Curitiba, Ponta Grossa and Guarapuava city regions. Populations of *S. lachnostachys* occur mainly on the Curitiba and Ponta Grossa plateaus. Three populations *S. lachnostachys* were chosen to represent the restricted distribution (Table 3, Figure 5). Our choice was based on the geographic distribution and topographic features. A voucher specimen for each population was deposited in UPCB Herbarium, Federal University of Paraná, Curitiba, Brazil (Table 3).

**Table 3:** Location and samples size of three populations of *Salvia lachnostachys* Benth.

Location	Latitude S	Longitude W	Altitude (m)	Voucher Number	No. of Samples
Curitiba	25°30'22"	49°18'30"	932	E.P. Santos 1251	23
Palmeira	25°28'10"	49°48'05"	1069	E.P. Santos 1264	24
São Luiz do Purunã	25°28'13"	49°39'33"	1089	E.P. Santos 1266	26

Leaves of 73 individuals were collected and stored in CTAB gel (3% Cationic Hexadecyl Trimethyl Bromide, 35% NACl). Genomic DNA was extracted using the modified 2× CTAB method by Doyle and Doyle. DNA integrity was examined in 1% agarose gel buffered with 1× TBE (tris-borate-EDTA buffer). Quality and concentration was doubled checked with a fluorimeter and a mass ladder comparison. Samples were kept in −20 °C until ISSR PCR processing.

### 4.2. ISSR-PCR Amplification

Nine previously tested primers for *S. lachnostachys* with good repetition and distinct polymorphic bands were chosen based on the existing literature (Table 1). ISSR-PCR reactions were carried out in a final 20 µL reaction volume containing 1.5 mM MgCl_2_, 0.2 mM of each dNTP, 2 µL of 10× TAQ buffer (Fermentas, Vilnius, Lithuania), 0.6 µM primer, 2% DMSO (dimethylsulphoxide), 1U Taq DNA polymerase, and 1–2 ng/µL of template DNA. Amplification was performed in an Mastercycler Gradient (Eppendorf, Hamburg, Germay) under the following conditions: initial denaturation at 94 °C for 2 min; then 35 cycles of 94 °C for 1 min, annealing at optimal temperature (Table 1) for 45 s, and 72 °C for 2 min; and a final extension at 72 °C for 7 min. The negative control used for each amplification consisted of a regular reaction tube without template DNA. In this case, the template DNA was replaced by an equal volume of ddH_2_O. PCR products were separated by electrophoresis using 1.5% agarose gel in 1× TAE (Tris-acetate-EDTA buffer), visualized with 4% ethidium bromide under ultraviolet light and photographed by 2UV Benchtop Transilluminator (UVP, Ultra-Violet Products Ltd., Cambridge, UK).

### 4.3. Data Analysis

ISSR data are dominant and, therefore, each band represents the genotype at a single bi-allelic locus. Samples were processed in three independent amplifications for each primer. All the electrophoresis photographic files were electronically analyzed (GelAnalyzer). Average bands size was computed and homology was inferred when size values were within a 2% range of error (considering their assigned size). Only fragments in the size range of 220 bp to 2.2 kb were included. The phenotypic pattern of the 73 individuals for all primers was recorded in a single binary matrix.

Sampling variances were examined to determine how many markers would be required for a given level of precision in the estimate of genetic distances [36,37]. The procedure was done within the software GENES version 2009.7.0 [38] using 1 to 159 markers and considering similarity under Nei and Li [39]. Ideal number of polymorphic bands was considered based on the suggested <0.05 stress value [40].

Simpson’s index was calculated by using the following formulas: S=Sum(1−sum f^2 )/N . Genetic variability within and among populations was evaluated using the percentage of polymorphic bands (*PPB*), observed number of alleles (*Ao*), effective number of alleles (*Ae*), Nei’s gene diversity (*He*) and Shannon’s information index (*I*) using PopGene version 3.2 [41]. Estimates of gene flow (*Nm*) were calculated following McDermott and McDonald [42]. Analysis of molecular variance (AMOVA) was used to estimate the partitioning of genetic variance among and within populations from different geographical origin with GenAlEx 6.4 software [43] and Arlequin version 3.11 [44]. Genetic relationships between populations were calculated using Nei’s unbiased genetic distance (D). The cluster analysis of different geographical groups was carried out using Unweighted Pair-group Method using Arithmetic average (UPGMA), and the dendrogram was constructed by the programs NTSYSpc [45] and PAUP*4b10 [46]. As gene substitution rate is not constant, we also implemented a parsimony heuristic search. To visualize genetic relationships among all ISSR phenotypes, their pairwise Euclidean distances were subjected to principal components analysis (PCA) as implemented in NTSYSpc [45]. A Bayesian analysis of ISSR population structure was performed on the entire data set using the program STRUCTURE version 2.1 [47]. Assuming maximal 10 population clusters (K), 10 independent runs of K = 1–10 were performed at 10^5^ Markov chain Monte Carlo samplings after a burn-in period of 5 × 10^4^ iterations—following the program’s dominant marker settings, the ‘‘no admixture” model was used and uncorrelated allele frequencies among populations were assumed. The most likely number of clusters was estimated according to the model value (∆K) based on the second order rate of change, with respect to K, of the likelihood function, following the procedure described by Evanno *et al.* [48].

## 5. Conclusions

To effectively conserve genetic variability in a germplasm bank, priority should be given to both the clusters of populations in the Palmeira locality. The dendrogram and Bayesian analysis indicate that this population is related to samples from Curitiba and São Luiz do Purunã. As the genetic variability is higher within populations and differences among the three examined populations are low, diversity could be maximized by concentrating sampling within the population from Palmeira.
